# Cooperation creates selection for tactical deception

**DOI:** 10.1098/rspb.2013.0699

**Published:** 2013-07-07

**Authors:** Luke McNally, Andrew L. Jackson

**Affiliations:** 1 Department of Zoology, School of Natural Sciences, Trinity College Dublin, Dublin, Republic of Ireland; 2 Trinity Centre for Biodiversity Research, Trinity College Dublin, Dublin, Republic of Ireland; 3 Centre for Immunity, Infection and Evolution, School of Biological Sciences, University of Edinburgh, West Mains Road, Edinburgh EH9 3JT, UK

**Keywords:** reciprocity, Machiavellian intelligence, deceit, comparative analysis, primates

## Abstract

Conditional social behaviours such as partner choice and reciprocity are held to be key mechanisms facilitating the evolution of cooperation, particularly in humans. Although how these mechanisms select for cooperation has been explored extensively, their potential to select simultaneously for complex cheating strategies has been largely overlooked. Tactical deception, the misrepresentation of the state of the world to another individual, may allow cheaters to exploit conditional cooperation by tactically misrepresenting their past actions and/or current intentions. Here we first use a simple game-theoretic model to show that the evolution of cooperation can create selection pressures favouring the evolution of tactical deception. This effect is driven by deception weakening cheater detection in conditional cooperators, allowing tactical deceivers to elicit cooperation at lower costs, while simple cheats are recognized and discriminated against. We then provide support for our theoretical predictions using a comparative analysis of deception across primate species. Our results suggest that the evolution of conditional strategies may, in addition to promoting cooperation, select for astute cheating and associated psychological abilities. Ultimately, our ability to convincingly lie to each other may have evolved as a direct result of our cooperative nature.

## 1. Introduction

Though there are multiple routes through which cooperation can evolve [1,2], conditional cooperation has become a major focus for studies on the evolution of cooperation in taxa of relatively advanced cognitive capabilities, particularly primates, and especially humans. As a result, myriad mechanisms of conditional behaviour that facilitate the evolution of cooperation have been identified, including direct reciprocity [3,4], indirect reciprocity [5,6], generalized reciprocity [7,8], partner choice [9–11], punishment [12,13] and reward [14,15]. While the details of how these mechanisms favour the evolution of cooperation differ, they all share the implicit requirement of correlation between the behaviours of interacting individuals [16–18]. This correlation leads to cooperative individuals receiving more cooperation, less punishment and/or more rewards than cheats, thus giving them a fitness advantage.

For this correlation to arise, an individual's behaviour must be either directly or indirectly conditional on their partner's behaviour (e.g. by accounting for their past actions or recognizing some cue of their current intentions). Almost all models for the evolution of conditional cooperation implicitly assume that the past or current actions of others can be assessed accurately and that cheats act passively during this assessment (i.e. they do not conceal their cheating). One possible mechanism by which cheats could avoid detection is tactical deception—the misrepresentation of the state of the world to another individual [19–23]. If individuals can use tactical deception to avoid their cheating being detected (e.g. by misdirecting a social partner's attention or misrepresenting their past actions or current intentions), they could circumvent enforcement mechanisms, gaining a fitness benefit.

Of course, deception may have benefits in many spheres of animal behaviour other than cooperative interactions (e.g. mating behaviour, aggressive encounters, etc.). Regardless, the benefit of eliciting cooperation at lower cost may help select for tactical deception in species with more frequent, and more diverse, forms of cooperation. Here we will first present a theoretical model exploring the evolution of tactical deception as a strategy in cooperative interactions before presenting a comparative analysis to test the relationship between deception and cooperation in primates.

## 2. Game-theoretic model

We consider the scenario of an iterated prisoner's dilemma between pairs of individuals in an infinite, well-mixed population. In any given interaction, individuals can choose to cooperate, bestowing a fixed benefit *b* on their partner at a fixed cost *c* to themselves, or to defect, bestowing no benefit and paying no cost. Here we will consider only conditional strategies that modify their cooperation in response to their partner's cooperation, though the general logic of our model also applies to cooperation based on punishment or reward. For simplicity we will assume that individuals can take one of the three fixed strategies for this game: conditional cooperator (CC), tactical deceiver (TD) or honest defector (HD). CCs aim to cooperate only with other cooperative individuals and not cooperate with defectors, whereas HDs simply always defect. We assume that TDs always defect but attempt to hide that defection from others in some manner (e.g. by waiting until they are unobserved or manipulating their reputation by lying), and that this deception carries a cost *d*. We do not specify the mechanism (e.g. partner choice or some form of reciprocity) by which conditional cooperation is achieved, but assume that conditional cooperation is somewhat constrained by repetition probability (i.e. current behaviour is somehow based on previous partner behaviour) and/or individual cognition (mistakes are sometimes made) so that CCs will cooperate with HDs in proportion *s* of their interactions. As TDs attempt to conceal their defection, CCs fail to recognize them as defectors with probability *q*, meaning that a CC will cooperate with a TD in a proportion *q* + *s* – *qs* of their interactions. Following our assumptions the average payoffs per round (*π*_*i*_) for each of the three strategies are: *π*_CC_ = (*b – c*)*x*_CC_ – *c*(*q* + *s – qs*)*x*_TD_ – *csx*_HD_ for CCs; *π*_TD_ = *b*(*q* + *s* – *qs*)*x*_CC_ – *d* for TDs; and *π*_HD_ = *bsx*_CC_ for HDs, where *x_i_* denotes the current frequency of strategy *i* in the population.

**Figure 1.**
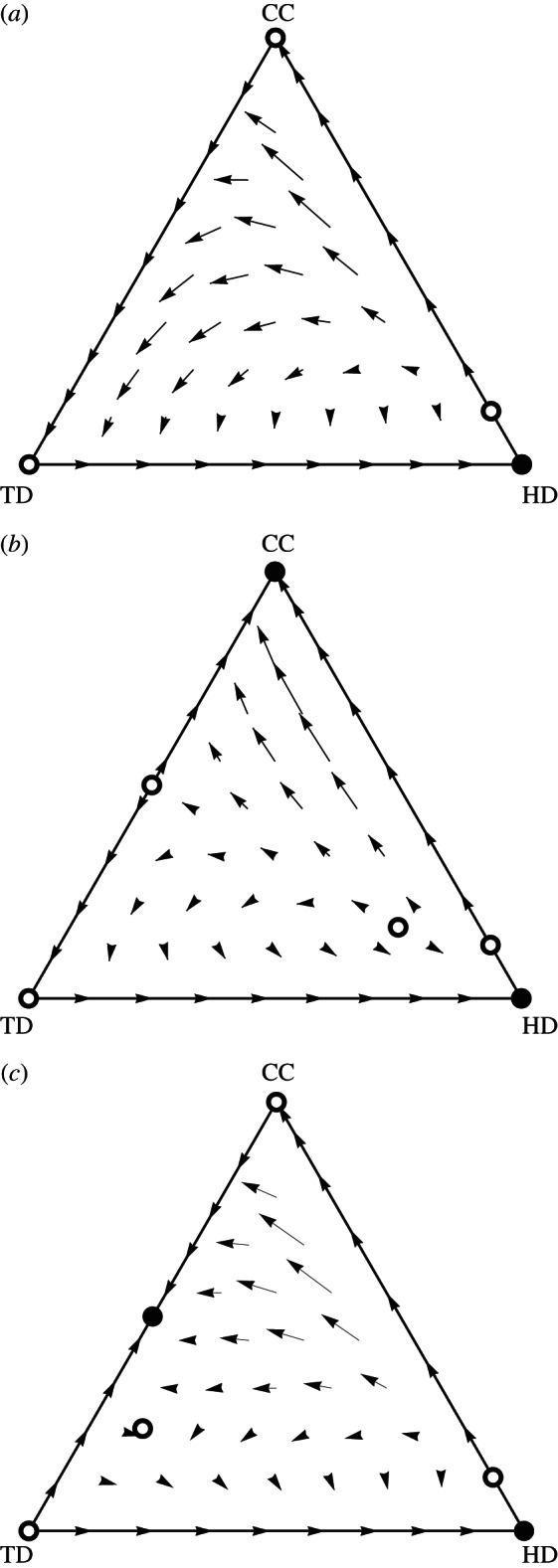
The dynamics of deception and cooperation. Shown are the evolutionary dynamics of the three strategies for the model with (*a,b*) constant and (*c*) negatively frequency-dependent efficiency of deception. Solid and open circles represent stable and unstable equilibria, respectively. Parameter values are *b* = 1.5, *c* = 0.5, *s* = 0.2 for all plots and (*a*) *q* = 0.8, (*b*) *q* = 0.5 and (*c*) *q* = 1 – *x*_TD_. Graphical output based on the Dynamo software [25].

Initially we will assume that *q* is a constant, but will later consider the case where *q* is frequency-dependent. We will constrain our analysis to the scenario where *b* – *c* > *sb* > 0, *q* > 0, *s* < 1 and *c* > *d* > 0, meaning that there is bistability between CCs and HDs (neither can invade the other from rarity), CCs do not dominate TDs and HDs dominate TDs (as TDs pay the cost of deception against HDs, while not receiving any benefit of eliciting cooperation). In this scenario, there are two qualitatively different outcomes possible, which are illustrated using the replicator equation *dx_i_*/*dt* = *x_i_*(*π*_*i*_ – *Σ_j_*π*_j_x_j_*) [24] in figure 1*a*,*b*. First, if *b*(*q* + *s – qs*) – *d* > *b* – *c*, then TDs dominate CCs. In this case, transient invasion of the TD strategy into a monomorphic population of CCs undermines the stability of the pure CC equilibrium, making honest defection the only Nash equilibrium and causing the collapse of cooperation (figure 1*a*). Beginning from a monomorphic population of CCs, a rare TD will invade and go to fixation. However, this equilibrium will be unstable and can be invaded by a rare HD (as *π*_TD_ = –*d* and *π*_HD_ = 0), which then goes to fixation. Alternatively, if *b*(*q* + *s – qs*) – *d* < *b – c*, then both conditional cooperation and honest defection are Nash equilibria and the introduction of TDs has little effect (figure 1*b*), as they cannot invade either monomorphic population. Note that, in both scenarios, there will be no stable mixed equilibrium owing to the lack of any negative frequency dependence among strategies.

**Figure 2.**
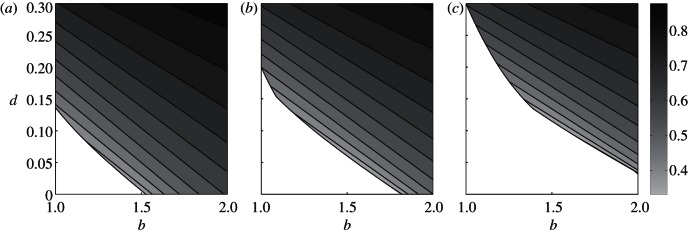
Equilibrium mixture of CCs and TDs. The coloured contour plot shows the frequency of CCs *x*_CC_* at the mixed equilibrium (frequency of TDs is 1 – *x*_CC_*) as a function of the benefit of cooperation *b* and the cost of deception *d* for the model with negative frequency dependence (see figure 1*c*). Darker (lighter) greys indicate a higher frequency of CCs (TDs) at the equilibrium. White areas indicate parameter values where there is no stable mixed equilibrium and the population converges on honest defection. Parameter values are *c* = 0.5, *q* = 1 – *x*_TD_ for all plots and (*a*) *s* = 0.1, (*b*) *s* = 0.2 and (*c*) *s* = 0.3.

In both of the cases above, TDs will only appear transiently before disappearing from the population, so how can TDs persist in the population? One possible factor leading to the coexistence of TDs and CCs is frequency dependence in the efficiency of deception. It would be expected that, as TDs become more common in the population, CCs would become more adept at spotting deception, such as via associative learning. The necessary negative frequency dependence for a mixed equilibrium may exist if the efficiency of deception *q* declines with increasing frequencies of TDs. Here we will consider the simplest scenario where *q* = 1 – *x*_TD_, so that deception is seldom detected when very rare, but is almost always detected when deception is the norm. In this scenario, a rare TD can always invade a monomorphic population of CCs as they will have payoffs *π*_TD_ = *b* – *d* and *π*_CC_ = *b – c*, and we have assumed that the cost of deception is less than that of cooperation (*d* < *c*). Additionally, a rare CC will be able to invade a population of TDs when –*cs* < –*d*. This negative frequency dependence can lead to a stable mixed equilibrium of *x*_CC_* CCs and 1 – *x*_CC_* TDs (figure 1*c*), which can resist invasion by HDs if *b*(1 – *s*)(*x*_CC_*)^2^ > *d*. The full expressions for the location and stability of this equilibrium are too unwieldy to yield analytical insight, but are numerically explored in figure 2. Extensive numerical exploration showed no other stable mixed equilibria. Increasing cost-to-benefit ratio (*c*/*b*) and decreasing costs of deception (*d*) increase the equilibrium frequency of TDs as their relative advantage over CCs is increased. However, TDs may become victims of their own success; if their equilibrium frequency becomes too high this equilibrium becomes invadable by HDs (i.e. *b*(1 – *s*)(*x*_CC_*)^2^ < *d*). Increasing the proportion of rounds CCs fail to defect against identified defectors (*s*) reduces the parameter space in which the mixed equilibrium is stable as the amount of cooperation TDs receive from CCs relative to that received by HDs is reduced (i.e. there is less benefit to outweigh the cost of tactical deception as conditional cooperation becomes less efficient).

## 3. Comparative analysis

Overall our theoretical results suggest that conditional cooperation can create selection pressures favouring investment in tactical deception. However, some form of negative frequency dependence in the efficacy of deception is necessary for tactical deception to be maintained at equilibrium. From these results, we can now ask what will happen as a species begins to cooperate in more ways (i.e. begin to play more games where some cooperation occurs). From our model we can predict that, given the requisite negative frequency dependence, adding more cooperative behaviours will lead to more tactical deception as conditional cooperation and tactical deception will coexist. As more cooperative behaviours (games) evolve, this is expected to increase selection for deception, which should be manifest as a positive relationship between the frequency of tactical deception and the number of cooperative behaviours a species engages in. In essence, new forms of cooperation could add additional dimensions of conditionality in the interactions among individuals, and these additional dimensions could potentially be exploited by deception.

To test this prediction, we used the collation of Byrne & Whiten [26] of the frequencies of tactical deception across non-human primates. This catalogue is based on a survey of expert researchers of a wide range of primate species and includes all records known at the time that conform to the following definition of tactical deception: ‘acts from the normal repertoire of the agent, deployed such that another individual is likely to misinterpret what the acts signify, to the advantage of the agent’ (p. 3 of [26]). While all forms of deception involve the misrepresentation of some information to other individuals, tactical deception implies that this misrepresentation is context-dependent and involves altering one's behaviour in a given context to mislead another individual. As did Byrne & Corp [27], we included all records that were considered by the original observers to meet this definition. This criterion allows the decision on whether tactical deception occurred to be made by the individuals who are best placed to do so: those who made the observations. As much research on tactical deception has occurred since the collation of Byrne & Whiten [26], we supplemented this collation with a literature search for examples of tactical deception. We searched ISI Web of Science using species names (and synonyms) and the term ‘deception’, and collated all examples where the authors believed that tactical deception occurred (references are included in the electronic supplementary material). Though these more recent examples of deception may suffer from additional biases to those in the collation of Byrne & Whiten (e.g. publication bias), they bring our dataset up to date with our current knowledge on deception in primates. We also note that all of our results hold qualitatively when only the collation of Byrne & Whiten is analysed. We performed two analyses on these frequencies of tactical deception across species. First, following Byrne & Corp [27], we analysed only observations of deception that were made on free-ranging individuals. However, as tactical deception may be more difficult to detect in the field, we also performed an analysis on all data including observations on captive individuals (as in [28]). We used the log of the deception frequency plus one in all analyses.

To assess how cooperativeness affects the frequency of tactical deception across species, we collated data on the presence of three cooperative behaviours that have been important during human evolutionary history: coalition formation, food sharing and alloparenting. We initially used data from the collations in [29–32], supplemented by additional searches for examples of these behaviours that were subsequently published. Searches were carried out using ISI Web of Science with search terms as follows: ‘coalition’, ‘alliance’, ‘ally’ and ‘agonistic support’ for coalition formation; ‘food sharing’ and ‘sharing’ for food sharing; ‘alloparental care’, ‘allomaternal care’, ‘alloparenting’ and ‘allonursing’ (including hyphenated variations) for alloparenting. The additional collated references are given in the electronic supplementary material (note that no additional examples of food sharing were found). We used the unweighted sum of the number of these cooperative behaviours a species is known to engage in, giving a score of cooperativeness between 0 and 3.

As deception has previously been shown to correlate with neocortex size [27] and loads strongly on measures of primate general intelligence [28], it is important to control for cognitive ability. To this end, we included neocortex ratio (size of the neocortex divided by the size of the rest of the brain) as a covariate in our analyses. Data on neocortex ratios were taken from multiple sources [33–36]. Volumes calculated using serial sections were supplemented with volumes calculated using magnetic resonance imaging for species where no serial section volume was available as these do not differ significantly within the primates [28]. We also performed an additional analysis using the log of the neocortex volume as a measure of cognitive capacity.

In total, data for all variables were available for 24 species spanning a wide taxonomic distribution: 3 prosimians, 7 New World monkeys, 10 Old World monkeys and 4 apes (data are given in the electronic supplementary material). Analyses were performed using phylogenetic generalized least-squares regression (PGLS) in R [37] with the caper package [38]. As estimation of the level of phylogenetic signal in a dataset becomes unreliable at small sample sizes [39,40], we set Pagel's *λ* = 1, which is equivalent to independent contrasts. We used version 3 of the consensus primate tree from the 10k Trees Project [41] to control for the phylogenetic relationships among species.

As the number of reports of deception may be influenced by the research effort a species receives, we included a measure of research effort in both analyses. We measured the research effort for all species as the number of papers on a species indexed in ISI Web of Science in the behavioural sciences, psychology, ecology, evolutionary biology, anthropology and zoology, between 1982 and 2012. We included the log of research effort as a covariate in our models.

**Figure 3.**
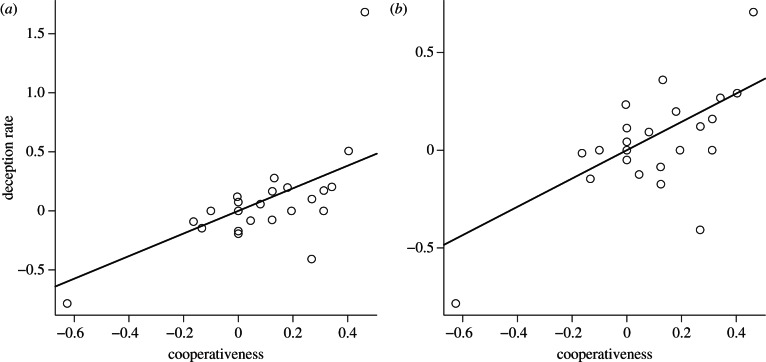
The relationship between deception and cooperation in non-human primates. The data points are independent contrasts for the rate of deception and cooperativeness scores with the effects of neocortex ratio and research effort partialled out for the (*a*) free-ranging and (*b*) full datasets. Lines are the predicted values from the models reported in the main text.

Our first analysis using neocortex ratio as a measure of cognitive capacity (model structure: log(deception rate + 1) ∼ cooperativeness + neocortex ratio + log(research effort)) showed a significant positive effect of cooperativeness on the rate of tactical deception for both the free-ranging only (*β* = 0.96, s.e. = 0.34, *p* = 0.010) and full (*β* = 0.72, s.e. = 0.23, *p* = 0.005) datasets (see figure 3). The neocortex ratio had no significant effect on tactical deception in the free-ranging dataset (*β* = 0.83, s.e. = 0.63, *p* = 0.20) or the full dataset (*β* = –0.039, s.e. = 0.43, *p* = 0.93). Our second analysis using the log of the neocortex volume as a measure of cognitive capacity (model structure: log(deception rate + 1) ∼ cooperativeness + log(neocortex volume) + log(research effort)) showed a significant positive effect of cooperativeness on the rate of tactical deception for both the free-ranging only (*β* = 0.97, s.e. = 0.36, *p* = 0.013) and full (*β* = 0.69, s.e. = 0.23, *p* = 0.007) datasets. Neocortex volume had no significant effect on tactical deception in the free-ranging dataset (*β* = 0.34, s.e. = 0.72, *p* = 0.64) or the full dataset (*β* = 0.32, s.e. = 0.47, *p* = 0.51).

## 4. Discussion

Both our theoretical model and the results of our comparative analysis provide strong support for the hypothesis that the presence of conditional mechanisms to enforce cooperation provides a major selective benefit to tactical deception. The explanation for this benefit is simple: tactical deception can allow individuals to elicit cooperation (or avoid punishment/gain reward) without paying the costs of cooperation. While there is much debate surrounding the proximate mechanisms involved in tactical deception, it has often been suggested to involve complex psychological abilities such as second-order intentionality and perspective taking [20–22,27,42]. While the taxonomic distribution of these complex proximate mechanisms is highly debatable, our results suggest that, at least in humans, conditional cooperation may have driven the development of many of our other complex psychological abilities by creating selection pressures favouring tactical deception, in addition to selecting for cooperative cognitive capacities such as shared intentionality [43].

Caution must be taken, however, when interpreting the results of our comparative analyses. The collation of tactical deception data of Byrne & Whiten [26] and our additional collations are based almost exclusively on anecdotal accounts of deception, rather than any systematic study. As such they are open to many potential conscious and unconscious biases during observation, and our comparative analyses should be seen as initial support of our hypothesis, rather than an authoritative test. Similarly, our collation of data on cooperation in primates also suffers from potential biases. The treatment of cooperative behaviours as present or absent has obvious pitfalls, and quantitative data are sorely needed. Limited research resources and the difficulty of publishing negative results may exacerbate this problem, though the inclusion of research effort in our analyses should ameliorate these effects to some extent. Further experiments and observational studies of both humans and non-human primates are necessary to ascertain the link between cooperation and tactical deception. These points also highlight important implications of our results for future empirical work on the phylogenetic distribution of deception and the proximate mechanisms underlying it. Deception is notoriously difficult to identify, particularly in the field, and our results suggest that focusing observations on more cooperative species and on individuals during cooperative exchanges may allow for the acquisition of greater volumes of data. Additionally, our model predicts that more costly forms of cooperative behaviour should show higher frequencies of deception, which could be tested by future observational and experimental studies. In addition to testing the evolutionary function of deception, such studies could help increase the amount of data on deception that can be collected with limited resources.

It is also worth noting that not all forms of deception are necessarily deliberate, and deliberate deception is likely to be very rare in non-human species. Individuals need not hold any beliefs regarding the effects of their actions in order to be deceptive. Alternatively, individuals could hold false beliefs about the consequences of their actions in order to deceive others, as has been suggested by Trivers in his theory of the evolution of self-deception in humans [44–48]. The essence of this theory is that the deliberate deception seen in humans has a high ‘cognitive load’, leading to tell-tale signs that an individual is deceiving you, such as signs of nervousness. By reducing this cognitive load, self-deception may allow deception of others to go unnoticed. Additionally, non-deliberate deception may not receive the same retribution as deliberate deception, as it may appear to be an ‘accident’ or owing to ignorance. Further empirical research with humans is necessary to ascertain the relative roles of deliberate and non-deliberate deception in eliciting cooperation from others. If Trivers’s thesis holds, the ability to elicit cooperation (or avoid punishment/gain reward) without paying the costs of cooperation may have also been a major selective factor in the evolution of self-deception in humans. However, it is important to stress that for functional tactical deception to occur an individual need not necessarily hold any belief regarding the effect of their actions. Thus, a lack of deliberate deception does not imply that self-deception is occurring.

While our simple model provides a general overview of the coevolution of deception and conditional cooperation, much further theoretical investigation of this relationship is required. Our model does not explicitly account for the underlying behavioural dynamics of conditional cooperation, and there is great potential for more mechanistic models. The inclusion of the possibility of tactical deception could lead to greater nuance in strategy evolution, with individuals showing different responses to the detection of honest cheating than to attempted deception. Given the infinite strategy space and complex dynamics of iterated games, and the additional complexity of the possibility of deception, approaches based on finite state automata [49–51] and/or artificial neural networks [52–54] may be particularly fruitful. Additionally, models of cognitive arms races [55] between TDs and CCs owing to selection for more subtle forms of deception and its detection could help shed further light on the drivers and evolutionary history of human cognitive evolution [20,56]. Additionally, it is worth noting some similarity between our results and those in signalling theory. It is a well-known result from signalling theory that dishonesty can only exist in the context of honesty [57]. Indeed conditional cooperation could be understood as a form of honest signalling, pointing to interesting parallels with the mechanisms we explore, which are worthy of further investigation.

Great progress has been made in recent years in elucidating both the proximate psychology of, and the ultimate explanations for, human cooperation. However, human psychology shows a mixture of conformance to moral/social norms that favour cooperation [58] and Machiavellian capabilities that facilitate selfish ends [20]. Explaining how evolutionary forces have balanced these tendencies is a major theoretical and empirical challenge. Our results suggest that studying the evolution of deception in the context of social interactions could provide a key window into the origins of this balance. Ultimately, this most Machiavellian element of human behaviour may be the product of one of our most beneficent characteristics—our tendency to seek mutually cooperative relationships.

## References

[1] Lehmann L Keller L 2006 The evolution of cooperation and altruism – a general framework and a classification of models. J. Evol. Biol. 19, 1365–1376 10.1111/j.1420-9101.2006.01119.x (doi:10.1111/j.1420-9101.2006.01119.x) 16910958

[2] West SA Griffin AS Gardner A 2007 Evolutionary explanations for cooperation. Curr. Biol. 17, R661–R672 10.1016/j.cub.2007.06.004 (doi:10.1016/j.cub.2007.06.004) 17714660

[3] Trivers RL 1971 The evolution of reciprocal altruism. Q. Rev. Biol. 46, 35–57 10.1086/406755 (doi:10.1086/406755)

[4] Axelrod R Hamilton WD 1981 The evolution of cooperation. Science 211, 1390–1396 10.1126/science.7466396 (doi:10.1126/science.7466396) 7466396

[5] Alexander RD 1987 The biology of moral systems. New York, NY: Aldine de Gruyter

[6] Nowak MA Sigmund K 1998 Evolution of indirect reciprocity by image scoring. Nature 393, 573–577 10.1038/31225 (doi:10.1038/31225) 9634232

[7] Pfeiffer T Rutte C Killingback T Taborsky M Bonhoeffer S 2005 Evolution of cooperation by generalized reciprocity. Proc. R. Soc. B 272, 1115–1120 10.1098/rspb.2004.2988 (doi:10.1098/rspb.2004.2988) PMC1559812 16024372

[8] Barta Z McNamara JM Huszár DB Taborsky M 2011 Cooperation among non-relatives evolves by state-dependent generalized reciprocity. Proc. R. Soc. B 278, 843–848 10.1098/rspb.2010.1634 (doi:10.1098/rspb.2010.1634) PMC3049049 20861047

[9] Roberts G 1998 Competitive altruism: from reciprocity to the handicap principle. Proc. R. Soc. Lond. B 265, 427–432 10.1098/rspb.1998.0312 (doi:10.1098/rspb.1998.0312)

[10] Sheratt TN Roberts G 1998 The evolution of generosity and choosiness in cooperative exchanges. J. Theor. Biol. 193, 167–177 10.1006/jtbi.1998.0703 (doi:10.1006/jtbi.1998.0703) 9689952

[11] McNamara JM Barta Z Fromhage L Houston AI 2008 The coevolution of choosiness and cooperation. Nature 451, 189–192 10.1038/nature06455 (doi:10.1038/nature06455) 18185587

[12] Boyd R Richerson PJ 1992 Punishment allows the evolution of cooperation (or anything else) in sizable groups. Ethol. Sociobiol. 13, 171–195 10.1016/0162-3095(92)90032-Y (doi:10.1016/0162-3095(92)90032-Y)

[13] Gardner A West SA 2004 Cooperation and punishment, especially in humans. Am. Nat. 164, 753–764 10.1086/425623 (doi:10.1086/425623) 29641920

[14] Oliver P 1980 Rewards and punishments as selective incentives for collective action: theoretical investigations. Am. J. Sociol. 85, 1356–1375 10.1086/227168 (doi:10.1086/227168)

[15] Rand DG Dreber A Ellingsen T Fudenberg D Nowak MA 2009 Positive interactions promote public cooperation. Science 325, 1272–1275 10.1126/science.1177418 (doi:10.1126/science.1177418) 19729661 PMC2875121

[16] Frank SA 1998 Foundations of social evolution. Princeton, NJ: Princeton University Press

[17] Woodcock S Heath J 2002 The robustness of altruism as an evolutionary strategy. Biol. Phil. 17, 567–590 10.1023/A:1020598804674 (doi:10.1023/A:1020598804674)

[18] Henrich J 2004 Cultural group selection, coevolutionary processes and large-scale cooperation. J. Econ. Behav. Organ. 53, 3–35 10.1016/S0167-2681(03)00094-5 (doi:10.1016/S0167-2681(03)00094-5)

[19] Whiten A Byrne RW 1988 Tactical deception in primates. Behav. Brain. Sci. 11, 233–273 10.1017/S0140525X00049682 (doi:10.1017/S0140525X00049682)

[20] Byrne RW Whiten A 1988 Machiavellian intelligence. Oxford: Oxford University Press

[21] Byrne RW Whiten A 1991 Computation and mindreading in primate tactical deception. In Natural theories of mind (ed. Whiten A), pp. 128–141 Oxford, UK: Blackwell

[22] Byrne RW Whiten A 1992 Cognitive evolution in primates: evidence of tactical deception. Man 27, 609–627 10.2307/2803931 (doi:10.2307/2803931)

[23] Lewis M Carolyn S 1993 Lying and deception in everyday life. London, UK: Guilford

[24] Weibull JW 1995 Evolutionary game thoery. Cambridge, MA: MIT Press

[25] Sandholm WH Dokumaci E Franchetti F 2012 Dynamo: diagrams for evolutionary game dynamics See http://www.ssc.wisc.edu/~whs/dynamo

[26] Byrne RW Whiten A 1990 Tactical deception in primates: the 1990 database. Prim. Rep. 27, 1–101

[27] Byrne RW Corp N 2004 Neocortex size predicts deception rate in primates. Proc. R. Soc. Lond. B 271, 1693–1699 10.1098/rspb.2004.2780 (doi:10.1098/rspb.2004.2780) PMC1691785 15306289

[28] Reader SM Hager Y Laland KN 2011 The evolution of primate general and cultural intelligence. Phil. Trans. R. Soc. B 366, 1017–1027 10.1098/rstb.2010.0342 (doi:10.1098/rstb.2010.0342) 21357224 PMC3049098

[29] Plavcan JM van Schaik CP Kappeler PM 1995 Competition, coalitions and canine size in primates. J. Hum. Evol. 28, 245–276 10.1006/jhev.1995.1019 (doi:10.1006/jhev.1995.1019)

[30] Jaeggi AV van Schaik CP 2011 The evolution of food sharing in primates. Behav. Ecol. Sociobiol. 65, 2125–2140 10.1007/s00265-011-1221-3 (doi:10.1007/s00265-011-1221-3)

[31] Riedman ML 1982 The evolution of alloparental care and adoption in mammals and birds. Q. Rev. Biol. 57, 405–435 10.1086/412936 (doi:10.1086/412936)

[32] Schoof VAM Jack KM Isbell LA 2009 What traits promote male parallel dispersal in primates? Behaviour. 146, 701–726 10.1163/156853908X399086 (doi:10.1163/156853908X399086)

[33] Stephan H Frahm H Baron G 1981 New and revised data on volumes of brain structure in insectivores and primates. Folia. Primatol. 35, 1–29 10.1159/000155963 (doi:10.1159/000155963) 7014398

[34] Zilles K Rehkamper G 1988 The brain, with special reference to the telencephalon. In Orang-utan biology (ed. Schwartz JH), pp. 157–176 Oxford, UK: Oxford University Press

[35] MacLeod CE Zilles K Schleicher A Rilling JK Gibson KR 2003 Expansion of the neocerebellum in hominoidea. J. Hum. Evol. 44, 401–429 10.1016/S0047-2484(03)00028-9 (doi:10.1016/S0047-2484(03)00028-9) 12727461

[36] Bush EC Allman JM 2004 The scaling of frontal cortex in primates and carnivores. Proc. Natl Acad. Sci. USA. 101, 3962–3966 10.1073/pnas.0305760101 (doi:10.1073/pnas.0305760101) 15007170 PMC374352

[37] R Core Development Team 2012 R: a language and environment for statistical computing. Vienna, Austria: R Foundation for Statistical Computing

[38] Orme D Freckleton R Thomas G Petzoldt T Fritz S Nick I 2012 Caper: comparative analyses of phylogenetics and evolution in R (v. 0.5) See http://CRAN.R-project.org/package=caper

[39] Freckleton RP Harvey PH Pagel M 2002 Phylogenetic analysis of comparative data: a test and review of evidence. Am. Nat. 160, 712–726 10.1086/343873 (doi:10.1086/343873) 18707460

[40] Münkenmüller T Lavergne S Bzeznik B Dray S Jombart T Schiffers K Thuiller W 2012 How to measure and test phylogenertic signal. Methods. Ecol. Evol. 3, 743–756 10.1111/j.2041-210X.2012.00196.x (doi:10.1111/j.2041-210X.2012.00196.x)

[41] Arnold C Matthews LJ Nunn CL 2010 The 10k Trees website: a new online resource for primate phylogeny. Evol. Anthropol. 19, 114–118 10.1002/evan.20251 (doi:10.1002/evan.20251)

[42] Premack DG Woodruff G 1978 Does the chimpanzee have a theory of mind? Behav. Brain. Sci. 1, 515–526 10.1017/S0140525X00076512 (doi:10.1017/S0140525X00076512)

[43] Moll H Tomasello M 2007 Coopeation and human cognition: the Vygotskian intelligence hypothesis. Phil. Trans. R. Soc. B 362, 639–648 10.1098/rstb.2006.2000 (doi:10.1098/rstb.2006.2000) 17296598 PMC2346522

[44] Trivers R 1991 Deceit and self-deception: the relationship between communication and consciousness. In Man and beast revisited (eds Robinson M Tiger L), pp. 175–191 Washington, DC: Smithsonian

[45] Trivers R 2000 The elements of a scientific theory of self-deception. Ann. NY Acad. Sci. 907, 114–131 10.1111/j.1749-6632.2000.tb06619.x (doi:10.1111/j.1749-6632.2000.tb06619.x) 10818624

[46] Trivers R 2010 Deceit and self-deception. In Mind the gap (eds Kappeler PM Silk J), pp. 373–394 Berlin, Germany: Springer

[47] Von Hippell B Trivers R 2011 The evolution and psychology of self-deception. Behav. Brain. Sci. 34, 1–56 10.1017/S0140525X10001354 (doi:10.1017/S0140525X10001354) 21288379

[48] Trivers R 2011 Deceit and self-deception: fooling ourselves the better to fool others. London, UK: Penguin

[49] Rubinstein A 1986 Finite automata play the repeated prisoner's dilemma. J. Econ. Theor. 39, 83–96 10.1016/0022-0531(86)90021-9 (doi:10.1016/0022-0531(86)90021-9)

[50] Ho TH 1996 Finite automata play repeated prisoner's dilemma with information processing costs. J. Econ. Dynam. Control. 20, 173–207 10.1016/0165-1889(94)00848-1 (doi:10.1016/0165-1889(94)00848-1)

[51] van Veelen M García J Rand DG Nowak MA 2012 Direct reciprocity in structured populations. Proc. Natl Acad. Sci. USA 25, 9929–9934 10.1073/pnas.1206694109 (doi:10.1073/pnas.1206694109) 22665767 PMC3382515

[52] Harald PG Fogel DB 1996 Evolving continuous behaviour in the iterated prisoner's dilemma. Biosystems 37, 135–145 10.1016/0303-2647(95)01550-7 (doi:10.1016/0303-2647(95)01550-7) 8924632

[53] Mitri S Floreano D Keller L 2009 The evolution of information suppression in communicating robots with conflicting interests. Proc. Natl Acad. Sci. USA 106, 15 786–15 790 10.1073/pnas.0903152106 (doi:10.1073/pnas.0903152106) PMC2747196 19805224

[54] McNally L Brown SP Jackson AL 2012 Cooperation and the evolution of intelligence. Proc. R. Soc. B 279, 3027–3034 10.1098/rspb.2012.0206 (doi:10.1098/rspb.2012.0206) PMC3385471 22496188

[55] Gavrilets S Vose A 2006 The dynamics of Machiavellian intelligence. Proc. Natl Acad. Sci. USA 103, 16 823–16 828 10.1073/pnas.0601428103 (doi:10.1073/pnas.0601428103) PMC1636539 17075072

[56] Dunbar RIM Schultz S 2007 Evolution in the social brain. Science 317, 1344–1347 10.1126/science.1145463 (doi:10.1126/science.1145463) 17823343

[57] Scott-Phillips TC Blythe RA Gardner A West SA 2012 How do communication systems emerge? Proc. R. Soc. B 279, 1943–1949 10.1098/rspb.2011.2181 (doi:10.1098/rspb.2011.2181) PMC3311886 22217724

[58] Fehr E Fischbacher U 2003 The nature of human altruism. Nature 425, 785–791 10.1038/nature02043 (doi:10.1038/nature02043) 14574401

